# 16S rRNA gene V4 amplicon sequence data from *Acropora pulchra* tissue microbiome samples from Madura Island, Indonesia

**DOI:** 10.1128/mra.00552-26

**Published:** 2026-07-17

**Authors:** Dining Nika Alina, Buntora Pasaribu, Ismail Maqbul, Qurnia Wulan Sari, Rita Rachmawati

**Affiliations:** 1 Department of Marine Sciences, Faculty of Fisheries and Marine Sciences, Universitas Padjadjaran, Bandung, West Java, Indonesia; 2 Research Centre for Biota System, National Research and Innovation Agency, Cibinong, Bogor, West Java, Indonesia; 3 Research Center for Conservation of Marine and Inland Water Resources, National Research and Innovation Agency, Bogor, West Java, Indonesia; University of Southern California, Los Angeles, California, USA

**Keywords:** coral microbiome, *Acropora pulchra*, 16S rRNA gene, amplicon sequencing

## Abstract

We report 16S rRNA gene V4 amplicon sequence data from five *Acropora pulchra* tissue microbiome samples collected from Madura Island, Indonesia, in September 2020. Raw reads are generated on the Illumina NovaSeq 6000 platform, and processed operational taxonomic unit-based files are publicly available for future coral microbiome studies.

## ANNOUNCEMENT

Coral-associated microbial communities are integral components of the coral holobiont and linked to nutrient cycling, host development, immune function, and responses to environmental stress (1, 2). In addition, coral microbiomes may exhibit host-associated and species-specific community patterns, making taxon-resolved sequence resources valuable for comparative studies across coral species and habitats (3, 4). *Acropora pulchra* is a branching coral commonly found in shallow tropical reef environments and, therefore, relevant for host-associated microbiome studies in environmentally dynamic habitats. Here, we report a 16S rRNA gene amplicon sequence data set from tissue microbiomes of *Acropora pulchra* collected from Madura Island, Indonesia, in September 2020.

Coral fragments (2 to 3 cm) were collected from five different *A. pulchra* colonies at Madura Island, Indonesia (−6.88328384485996, 113.09042463031899). Coral tissue was detached from the skeleton using a modified air-blasting approach (5), rinsed with filtered ice-cold 1× phosphate-buffered saline to produce a slurry, centrifuged to obtain a tissue pellet, and stored at −20°C until DNA extraction. Microbial DNA was extracted using the DNeasy Blood and Tissue Kit (Qiagen, Hilden, Germany). The bacterial 16S rRNA gene V4 region was amplified with primers 515F (5′-GTGCCAGCMGCCGCGGTAA-3′) and 806Rb (5′-GGACTACHVGGGTWTCTAAT-3′) (6). PCR amplification was performed by Novogene, Singapore, using Phusion High-Fidelity PCR Master Mix (New England Biolabs) with cycling conditions of 98°C for 1 min, 30 cycles of 98°C for 10 s, 50°C for 30 s, and 72°C for 30 s, and 72°C for 5 min. Amplicon libraries were sequenced on the Illumina NovaSeq 6000 platform to generate paired-end 2 × 250 bp reads.

Initial read processing, including barcode assignment, paired-end merging, quality filtering, and chimera removal, was performed by Novogene. According to the vendor workflow, paired-end reads were merged using FLASH v1.2.7; quality filtering followed the QIIME v1.7.0 quality-control process; and chimeric sequences were identified with UCHIME against the Gold database and removed. The resulting clean data were then reanalyzed in QIIME 2 version 2022.11 (7). Single-end clean FASTQ files were imported into QIIME 2, and default parameters were used for all software unless otherwise specified. Because clean reads were used as input, no additional trimming or truncation was applied (trim-left 0 and trunc-len 0). Operational taxonomic units (OTUs) were generated by *de novo* clustering at 97% sequence identity using the q2-vsearch plugin, and taxonomic classification was performed against the SILVA v138 reference database using the q2-feature-classifier plugin (8).

**Fig 1:**
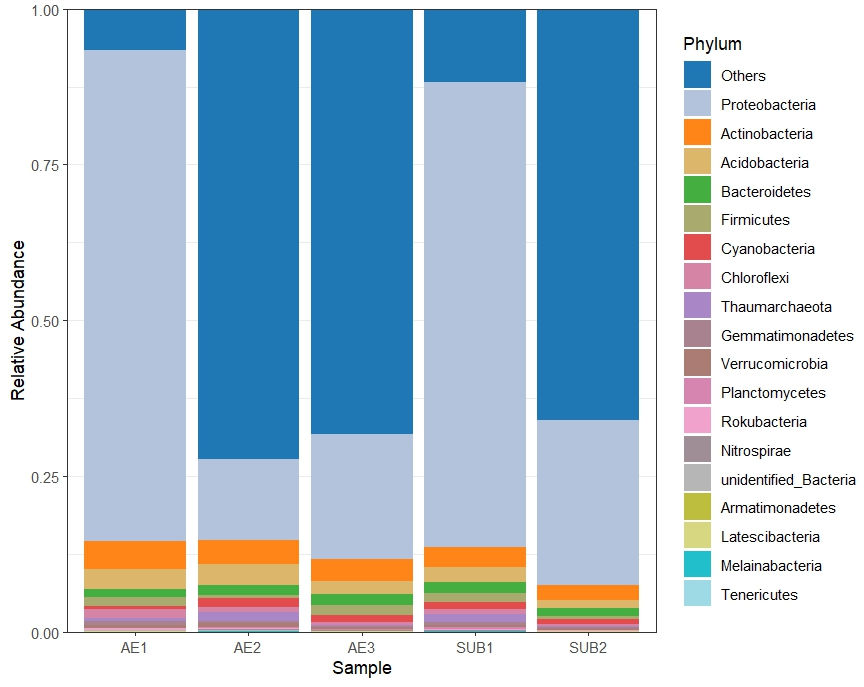
Phylum-level relative abundances across five *Acropora pulchra* tissue microbiome samples collected from Madura Island, Indonesia. Taxa with low relative abundance were grouped as “others.”

Six coral tissue samples were initially processed. One sample was excluded because resequencing did not resolve earlier quality-control concerns, and its OTU profile remained inconsistent with the other coral tissue samples. The final released data set, therefore, comprised five samples retained for downstream OTU clustering and taxonomic analysis. A summary of sample identifiers, SRA accession numbers, BioSample accession numbers, total reads, and OTU counts is provided in Table 1. At the phylum level, Proteobacteria dominated all five samples, and Actinobacteria and several low-abundance phyla were also detected (Fig. 1). This data set provides a publicly available coral microbiome resource from an Indonesian reef environment.

**TABLE 1:** Summary of the five *Acropora pulchra* tissue microbiome samples included in the released data set

Sample name	SRA accession no.	BioSample accession no.	Total reads	No. of OTUs
AE1	SRR38326629	SAMN57560821	174,147	3,970
AE2	SRR38326628	SAMN57560822	196,920	3,592
SUB1	SRR38326627	SAMN57560823	178,242	3,088
AE3	SRR38326626	SAMN57560824	174,159	3,856
SUB2	SRR38326625	SAMN57560825	190,139	3,181

## Data Availability

The raw 16S rRNA gene amplicon sequence data have been deposited in the National Center for Biotechnology Information (NCBI) Sequence Read Archive under BioProject accession number PRJNA1459641. The five released samples correspond to SRA run accession numbers SRR38326629, SRR38326628, SRR38326627, SRR38326626, and SRR38326625, and the associated BioSample accession numbers are listed in Table 1. Processed files, including the metadata table, OTU table, taxonomy table, and associated QIIME 2 outputs, are available in Zenodo at https://doi.org/10.5281/zenodo.19844137.
